# Fundamental Neurochemistry Review: Sphingolipids and Ceramides in Brain Development

**DOI:** 10.1111/jnc.70262

**Published:** 2025-10-21

**Authors:** Kaviya Chinnappa, Fiona Ballorin, Fiona Francis

**Affiliations:** ^1^ Inserm, CNRS, Center of Neuroscience Neuro‐SU, Sorbonne Université Paris France; ^2^ Inserm, CNRS, Institut de Biologie Paris‐Seine, IBPS, Sorbonne Université Paris France; ^3^ Institut du Fer à Moulin, Inserm, Sorbonne Université Paris France

**Keywords:** brain development, ceramides and sphingolipids, cerebral cortex, lipids, neurological disorders

## Abstract

Lipids are emerging as key players in regulating neural stem cells and their progeny. Recent lipidomic profiling studies clearly illustrate the significance of lipids across cerebral cortical development and evolution. In this review, we identify the existing knowledge concerning the importance of lipids in cortex development and neurogenesis processes, with a special emphasis on ceramide‐based sphingolipids. We then discuss the lipidation of factors that are relevant for cortex development and how their interaction with ceramides facilitates certain intracellular processes. We further summarize the importance of ceramides in different intracellular compartments and organelles, and finally discuss the alterations of sphingolipid metabolism in neurological disorders, particularly of neurodevelopmental origin.

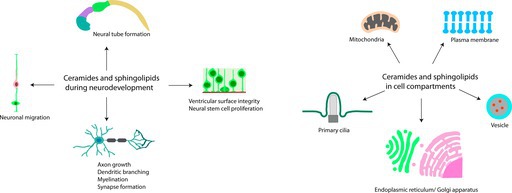

## Graphical abstract

*Ceramides and sphingolipids in brain development and cell compartments*. Ceramides and sphingolipids are known to influence multiple steps of brain development, including the formation of the neural tube, neural stem cell polarity and proliferation at the ventricular boundary, neuronal migration—for example, whilst migrating on radial glial processes, as well as during axon development, dendritic branching, myelination, and synapse formation. Ceramides are indeed known to be important in different cell compartments, including the plasma membrane, endoplasmic reticulum, Golgi apparatus, mitochondria, primary cilia, and vesicles. Describing these processes in detail, we also discuss disturbances in sphingolipid homeostasis, causing several neurological disorders.

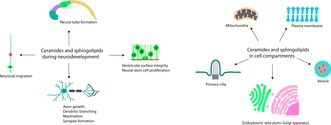

**Abbreviations**
AD: Alzheimer disease
aPKC: atypical protein kinase C
ASD: autism spectrum disorder
BLBP: brain lipid binding protein
BP: basal progenitor
bRG: basal radial glia
CDG: congenital disorder of glycosylation
Cers: ceramide synthase
CERT: ceramide transfer protein
CGT: UDP‐galactose: ceramide galactosyltransferase
CLNs: neuronal ceroid lipofuscinoses
CMs: malformations of cortical development
CNS: central nervous system
CSF: cerebrospinal fluid
CST: cerebroside sulfotransferase gene
dhS1P: dihydrosphingosine 1‐phosphate
E: embryonic day
EMT: epithelial to mesenchymal transition
ER: endoplasmic Reticulum
ES: embryonic stem cell
Fabp: fatty acid binding protein
FASN: fatty acid synthase
fln: Flincher
GCS: glucosylceramide synthase
GFAP: glial fibrillary acidic protein
GLAST: astrocyte specific glutamate transporter
GPI: glycosylphosphatidylinositol
ID: intellectual disability
IFT: intraflagellar transport
ISVZ: inner subventricular zone
LSD: lysosomal storage disorder
MAG: myelin associated glycoprotein
MDCK: Madin‐Darby Canine Kidney cells
MOGHE: mild malformation of cortical development with oligodendroglia hyperplasia in epilepsy
MOM: mitochondria outer membrane
NECs: neuroepithelial cells
NSC: neural stem cell
OSVZ: outer subventricular zone
PD: Parkinson disease
PME: progressive myoclonic epilepsy
PNS: peripheral nervous system
Porcn: Porcupine
PPP: pentose phosphate pathway
RG: radial glia
Shh: sonic hedgehog
SMase: sphingomyelinase
Sphk: sphingosine kinase
SPT: serine palmitoyltransferase
SVZ: sub‐ventricular zone
TKTL1: transketolase 1
to: toppler
Ugcg: UDP‐Glucose ceramide glucosyltransferase
UPR: unfolded protein response
VZ: ventricular zone
Wg: Wingless
Wls: Wntless

## 1 Introduction

### 1.1 Lipids in Brain Development and Function

Lipids are the major components of cellular membranes and are essential for the maintenance of structural integrity. They are also the substrates for metabolic fuel, they anchor membrane proteins, and in some cases can serve as precursors for a range of signaling molecules. The brain has the second‐highest lipid content after adipose tissue, accounting for 10%–12% of the fresh weight and 50% of its dry weight (Sastry 1985; Han 2007). It is mainly composed of cholesterol, phospholipids, and sphingolipids (Han 2007). Lipids hence have numerous roles in the brain.

Evidence for the isolation of sphingolipids from the brain stems from the late 19th century (1884) by Thudichum, who introduced the name “sphingosin”, named after the mythical sphinx, owing to their enigmatic nature. In later years, the chemical structure of sphingosines, the building blocks of all sphingolipids, was elucidated, followed by the discovery of different classes of complex sphingolipids, including sphingomyelins and glycosphingolipids (Thudichum 1962; Hannun and Obeid 2018). The class of complex glycosphingolipids, gangliosides, is particularly abundant in the brain, with 10‐ to 30‐fold more than any other tissue in the body. The dynamic expression of different gangliosides across developmental time and space in different cell types and in multiple species, including human, elucidates their importance during development and adult brain function (Hilbig et al. 1983; Svennerholm et al. 1989; Sonnino et al. 1990; Kracun et al. 1992; Kotani et al. 1993; Hirschberg et al. 1996; van Echten‐Deckert and Herget 2006; Ngamukote et al. 2007; Olsen and Færgeman 2017; Sipione et al. 2020; Vasques et al. 2023). While the gray matter of the brain is highly enriched in gangliosides, myelin and oligodendrocytes are enriched in sphingomyelin, galactosylceramide, and sulfatide (Olsen and Færgeman 2017; Poitelon et al. 2020). With this expression information, it has become crucial to further study the origin and function of sphingolipids during brain development.

### 1.2 Lipidomic Studies Across Brain Development and Evolution

Lipidomic profiling of mouse brain tissue across different ages between 4 and 52 weeks identified decreased mass percentages of total glycerophospholipids and increased mass percentages of total sphingolipids across time. It is also important that sphingolipids exhibit acyl chain length‐dependent changes with age, with longer acyl‐chain (ranging from C20 to C26) sphingolipids showing an increase and shorter acyl‐chain sphingolipids showing a decrease across time (Tu et al. 2018).

Lipidomic analyses of neural compared to non‐neural tissue from humans, chimpanzees, rhesus macaques, and mice revealed that among these species, the human brain had the most distinct lipid composition since 76% of the lipid compounds analyzed were either enriched or depleted compared to human non‐neural tissue. Thus, the largest divergence of lipidome between the neural and non‐neural tissues was detected in humans, followed by chimpanzees and macaques, with the smallest divergence in mice. It is further interesting to note that the concentration levels of lipids that are brain‐enriched evolve approximately four times faster among primates, showing further acceleration of change in human neocortical regions compared with lipids of non‐neural tissues. Parallel transcriptomic analyses identified relative expression changes of the enzymes responsible for the lipidome changes. Thus, lipid diversity and divergence most likely contribute to the evolution and functional complexity of the brain, especially the neocortex (Bozek et al. 2015).

Also, lipidomic analyses from macaques, chimpanzees, and humans identified significant concentration differences of various lipids between humans and chimpanzees or macaque brains. The greatest species‐specific differences were found to occur within the first 10 years of human life, with the lipidome of humans compared to chimpanzees showing an approximately two‐fold more distinct profile at 1 year of human age compared to adult stages. Lipidomic analyses of the human brain showed higher concentrations of specific lipid classes, including neutral glycosphingolipids, triacylglycerols, and acidic glycosphingolipids in the adult brain, whereas higher concentrations of ceramides were identified in infant brains (Q. Li et al. 2017).

Single cell lipidomic analyses of the human cortex across different developmental time points identified a significant increase in the number of lipids including ceramides and other complex sphingolipids during the peak periods of neurogenesis, suggesting their importance in early cortical development (Bhaduri et al. 2021). Measurement of different sphingolipids (including sphingomyelin, sphingosine, sphingosine 1‐phosphate, and ceramide) at different postnatal ages in rats also revealed a profound increase of all sphingolipids across postnatal brain development illustrating their structural importance during postnatal brain growth (Novgorodov et al. 2011).

Thus, the diversity and concentrations of neuro‐lipids, and their peaks during key developmental steps, especially the case for sphingolipids, all point to major roles in brain development.

### 1.3 Sphingolipid and Ceramide Biogenesis and Recycling

**FIGURE 1:**
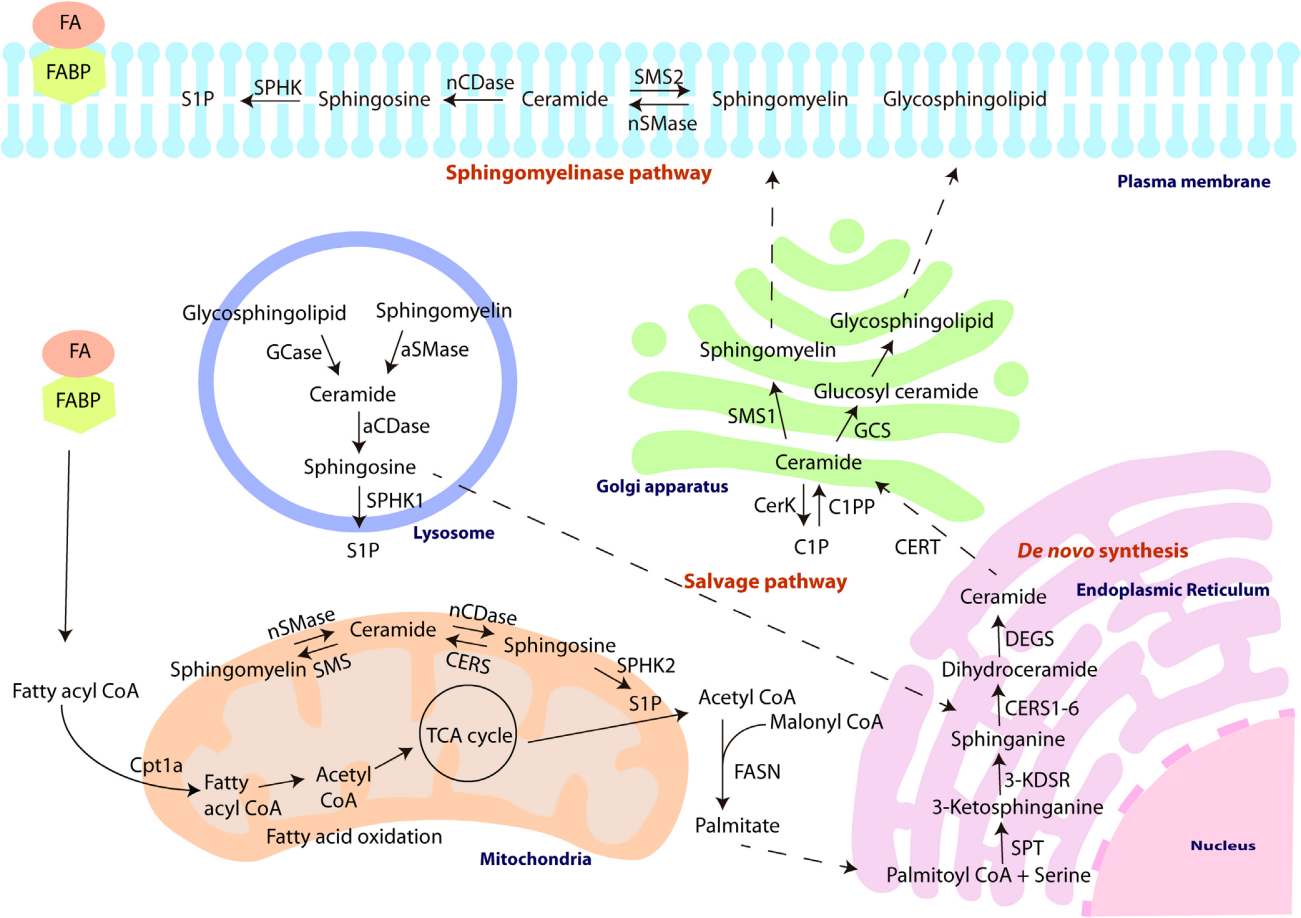
Sphingolipid biogenesis. FASN utilizes acetyl CoA derived from fatty acid oxidation (TCA cycle) and malonyl CoA to form palmitate. Palmitoyl CoA and Serine in the ER undergo a series of reactions to form ceramide through *de novo* biogenesis. Ceramide is transferred to the Golgi apparatus where it forms more complex sphingolipids, which are targeted to the plasma membrane. Sphingomyelin in the plasma membrane and in lysosomes is broken down to ceramide by the action of sphingomyelinases (SMases) by SMase pathways. Sphingosine produced from ceramide in lysosome is recycled back to the ER for ceramide biogenesis through the salvage pathway. 3‐KDSR, 3‐ketodihydrosphingosine reductase; aCDase, Acid ceramidase; aSMase, Acid sphingomyelinase; C1P, Ceramide 1‐phosphate; CERS, Ceramide synthase; CERT, Ceramide transfer protein; DEGS, Delta 4‐desaturase, sphingolipid; FASN, Fatty acid synthase; GCase, Glucosylceramidase; GCS, Glucosylceramide synthase; nCDase, Neutral ceramidase; nSMase, Neutral sphingomyelinase; S1P, Sphingosine 1‐phosphate; SMS, Sphingomyelin synthase; SPHK, Sphingosine kinase; SPT, Serine palmitoyltransferase.

Ceramides represent the building blocks of complex sphingolipids and are generated via three main pathways: the *de novo* pathway, the sphingomyelinase (SMase) pathway, and the salvage pathway (Harrison et al. 2018). As shown in Figure 1, the *de novo* pathway takes place primarily in the endoplasmic reticulum (ER) and begins with the condensation of serine and palmitoyl CoA by serine palmitoyltransferase (SPT) to produce 3‐ketodihydrosphingosine, which is then converted by 3‐ketodihydrosphingosine reductase to dihydrosphingosine (sphinganine). Ceramide synthase then acylates sphinganine to form dihydroceramide, which is desaturated by dihydroceramide desaturase to form ceramides (Menaldino et al. 2003). There are six different ceramide synthases in mammals that show specificity towards different subsets of acyl‐CoAs (Voelzmann and Bauer 2010). Ceramides, once produced, are then delivered to the Golgi apparatus by vesicular transport or translocation via the ceramide transfer protein (CERT) (Hanada 2010). Ceramides at the Golgi apparatus are further modified by the addition of head groups to form complex sphingolipids such as sphingomyelin and glycosphingolipids (Harrison et al. 2018). The formed lipids are then transported by vesicular transport or lipid‐transfer proteins to the plasma membrane (Samaha et al. 2019).

The SMase pathway produces ceramide at the plasma membrane or in lysosomes (neutral or acid SMase). Ceramides can be further catabolized to sphingosine and free fatty acids by the action of acid ceramidases in late endosomes and lysosomes (Pavoine and Pecker 2009). Ceramides can also be phosphorylated to ceramide 1‐phosphate by ceramide kinases and, similarly, sphingosine can be phosphorylated to sphingosine 1‐phosphate by sphingosine kinases, both of which act as bioactive lipids (Hait and Maiti 2017). Ceramides can also be generated by the action of ceramide synthases via the salvage pathway by the recycling of sphingosine produced in late endosomes and lysosomes and then processed in the ER (Kitatani et al. 2008).

Ceramides and sphingolipids, being abundant in the brain, must clearly influence brain development and function by acting as the structural components of lipid‐based cell membranes and rafts, facilitating protein–membrane and protein–protein interactions. They can also facilitate signal transduction or act as bioactive lipid signaling molecules themselves.

## 2 Cerebral Cortex Development, Neural Stem Cells (NSCs), Metabolism and Importance of Lipids

### 2.1 Cell Types and Key Functions

**FIGURE 2:**
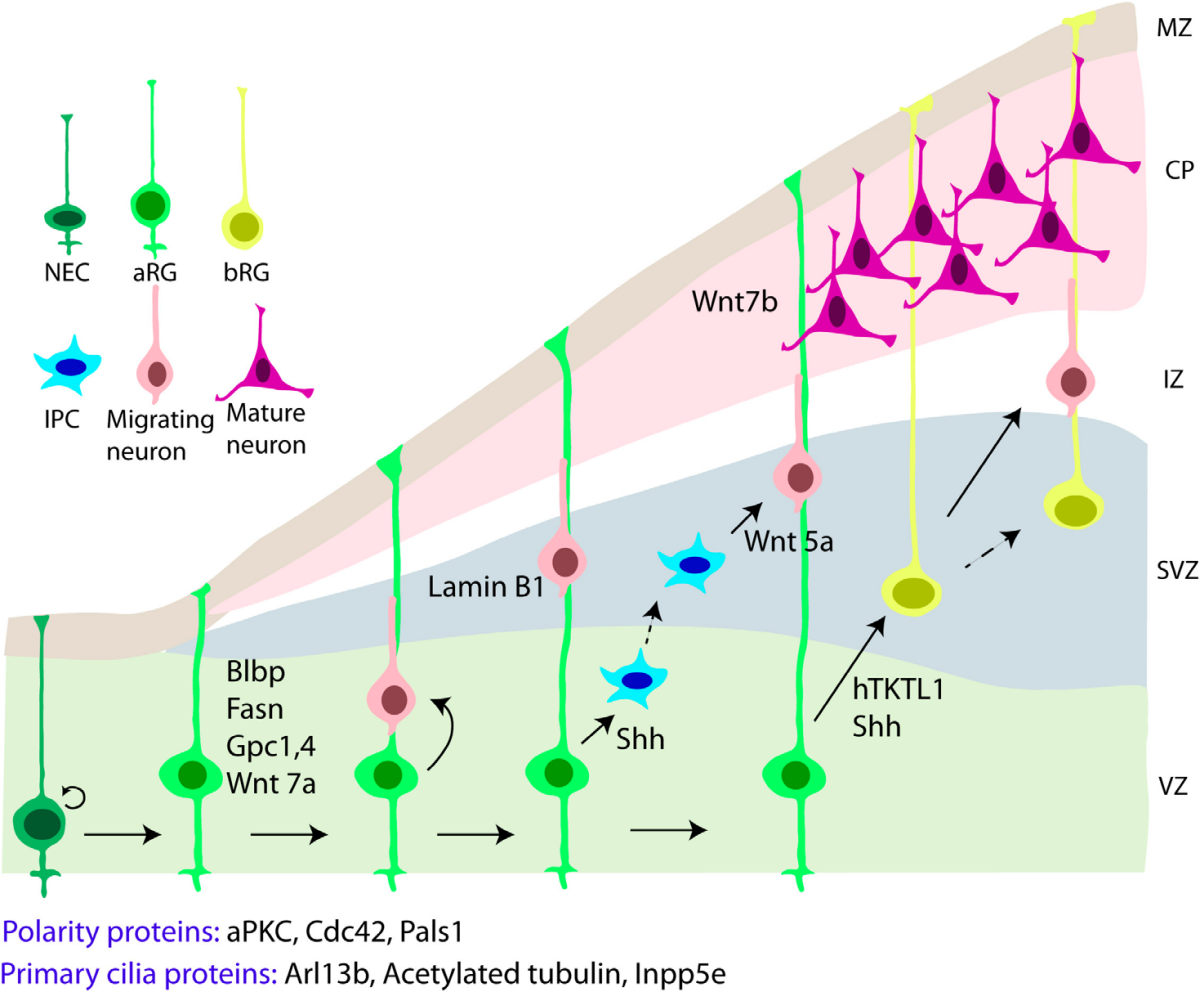
Lipid modified signaling proteins in cortex development. Corticogenesis begins with neuroepithelial cells (NECs) giving rise to apical RGs (aRGs), which divide further to form neurons or intermediate progenitor cells (IPCs), or basal radial glial cells (bRGs) that further form neurons. The newly formed neurons migrate along RG fibers and finally occupy the cortical plate. Involvement of several lipid forming/binding/modified proteins in this process is illustrated here. aRG, Apical radial glia; bRG, Basal radial glia; IPC, Intermediate progenitor cell. These cells can be collectively known as neural stem cells (NSCs). CP, cortical plate; IZ, intermediate zone; MZ, marginal zone; SVZ, subventricular zone; VZ, ventricular zone.

Neuroepithelial cells (NECs) represent primordial cells of cortex development forming the initial neuroepithelium. Although a single layer, the neuroepithelium appears multi‐layered (pseudostratified) as the cells undergo interkinetic nuclear migration, performing mitosis at the apical surface, with nuclei then ascending basally for S phase of the cell cycle (Takahashi et al. 1995). NECs, showing typical epithelial features, are highly polarized cells with their apical endfeet at the ventricular surface containing a primary cilium, a small signaling organelle exposed to cerebrospinal fluid (CSF), and basal endfeet touching the basal lamina at the surface of the brain. Tight and adherens junctions are characteristic features of NECs, present at the apical side of the lateral plasma membrane (Zhadanov et al. 1999; Manabe et al. 2002; Wodarz and Huttner 2003). These cells are highly proliferative, and with ongoing neurogenesis, they transform into more fate‐restricted progenitors, radial glial cells (RGs), upon downregulation of certain epithelial features such as tight junctions, as well as the appearance of astroglial features such as astrocyte‐specific glutamate transporter (GLAST), the Ca^2+^‐binding protein S100β, glial fibrillary acidic protein (GFAP), vimentin, and brain lipid binding protein (BLBP) (Hartfuss et al. 2001; Götz and Huttner 2005). This transition occurs in mouse around embryonic day 10 (E10). RGs, whose soma occupy a ventricular zone (VZ), maintain apical‐basal polarity and also undergo interkinetic nuclear migration. Centrosome and primary cilia biogenesis at the apical surface are tightly linked to the cell cycle. RGs undergo symmetric divisions to self‐renew and asymmetric divisions to form also basal or intermediate progenitors (BPs or IPCs) and neurons (Götz and Huttner 2005) (Figure 2). BPs are multipolar cells forming the sub‐VZ (SVZ), where they normally undergo symmetric divisions in mouse to form neurons (Kowalczyk et al. 2009). Another kind of highly proliferative BP is called a basal radial glial cell (bRG) whose soma is found above the VZ; these cells usually continue to exhibit a long basal process. bRGs are present in abundant amounts in gyrencephalic brain species, including humans, but are less prevalent in mouse (Hansen et al. 2010; Reillo et al. 2011). They help form an extended SVZ in gyrencephalic species, further divided into inner and outer SVZ (ISVZ and OSVZ). Notably, the abundance of OSVZ progenitors has been linked to the degree of cortical folding (gyrification index) across different species (Reillo et al. 2011; Del‐Valle‐anton and Borrell 2022). Neurons, once they are formed, migrate long distances along RG and bRG basal fibers and reach the developing cortical plate, settling in an inside‐out manner, with the earliest born neurons occupying the deep layers and later born neurons occupying the superficial layers (Sidman and Rakic 1973). This process is largely completed before birth. Once neurogenesis declines, the process of gliogenesis starts during late embryonic development (Sauvageot and Stiles 2002). Concerning cortical neurons, they grow axons and dendrites and make synaptic connections. Myelin surrounds many nerve axons in a process that occurs after birth. It helps to insulate axons and allows them to transmit action potentials efficiently (Fletcher et al. 2021). Although different extrinsic and intrinsic factors known to influence cortical development have been extensively studied, it remains greatly understudied how lipids and lipid binding/modified proteins influence these processes.

### 2.2 Fatty Acid Binding Proteins, Lipid Modifications and Signaling

#### 2.2.1 Fatty Acid Binding Proteins

The transport of fatty acids in aqueous cytoplasm has been shown to be mediated by fatty acid binding proteins (Fabps). Three of the ten *Fabps* identified in mammals, *Fabp3*, *Fabp5*, and *Fabp7*, are expressed in the developing and/or adult brain. Expression of *Fabp7* (also known as Blbp, Brain lipid binding protein) increases during embryonic mouse brain development, reaching its maximum during the peak period of neurogenesis at E14, and decreases significantly during postnatal stages (Feng et al. 1994; Kurtz et al. 1994; Owada et al. 1996). Expression of *Fabp5* mRNA is detected during mid‐neurogenesis in rat, reaches its peak at birth, and then gradually decreases during postnatal stages (Owada et al. 1996). *Fabp3* mRNA levels gradually increase after birth into adulthood (Owada et al. 1996). Thus, the expression patterns of *Fabp7* and *Fabp5* coincide with the neurogenesis period and *Fabp3* with synaptogenesis and myelinogenesis (Liu et al. 2010). *Fabp7* is specifically expressed in NECs and RGs (Feng et al. 1994; Kurtz et al. 1994; Owada et al. 1996; Götz and Huttner 2005). Analyses of *Fabp7* knockout (KO) mice illustrated the importance of *Fabp7* in neurogenesis and astrogenesis through the maintenance of the NSC pool (A. Watanabe et al. 2007). Importantly, expression of *Fabp7* is modulated by different factors and signaling pathways crucial for cortical development, such as Reelin‐Dab1, Notch, Pax6, and POU‐domain proteins (Josephson et al. 1998; Hartfuss et al. 2003; Anthony et al. 2005; Arai et al. 2005). Thus, fatty acids, upstream of sphingolipid generation, play a central role in influencing several key steps of cortical development.

#### 2.2.2 Lipid Modifications

Addition of lipid molecules to proteins helps to regulate protein trafficking, localisation, protein‐membrane interactions, protein–protein interactions, protein stability, and signal transduction (reviewed in detail in (Jiang et al. 2018; Yuan et al. 2024)). Lipid‐modified proteins are known to associate with lipid rafts enriched in cholesterol and sphingolipids (Melkonian et al. 1999). Common signaling molecules important for neurodevelopment, such as Wnt and Sonic hedgehog (Shh), were found to undergo several of these lipid modifications as part of their processing (Willert and Nusse 2012). We review these data here.

#### 2.2.3 Wnt Signaling

Wnt signaling is involved in the proliferation and differentiation of NSCs, multipolar to bipolar transitioning of neurons, their migration, and laminar fate specification (Woodhead et al. 2006; Munji et al. 2011; Harrison‐Uy and Pleasure 2012; Boitard et al. 2015; Bocchi et al. 2017) (Figure 2). Concerning lipid modifications, Wnt3a, regulating cell fate, proliferation, and differentiation, is acylated at Serine 209 (Janda et al. 2012; Gao and Hannoush 2014). Mutation of this S209 residue affected the secretion of the protein without affecting its stability (Takada et al. 2006). Similarly, palmitoylation of Wingless (Wg, C93), the fly homolog of Wnt proteins, has been shown to be important for both secretion and signaling activity, whereas acylation of Wg (S239) has been linked to signaling activity (Franch‐Marro et al. 2008). The ER resident protein Porcupine, which resembles membrane‐bound O‐acyl transferases, is presumed to catalyze the acylation process of Wnt (Takada et al. 2006). Deletion of mouse *Porcn* was found to abolish Wnt secretion, and mutations in its human ortholog lead to the condition of focal dermal hypoplasia (FDH) with developmental abnormalities (Barrott et al. 2011). Porcupine‐dependent lipidation of all Wnts except WntD is required for their interaction with the transmembrane receptor protein Wntless (Wls), which escorts it to the cell surface beyond the membrane tethering property of lipidation (Herr and Basler 2012). Studies by Coombs and colleagues propose that Wls binds Wnt through a lipid binding domain and that vacuolar acidification is essential to release lipidated Wnt3a from Wls in secretory vesicles and to facilitate the transfer of Wnt3a to a soluble carrier protein, possibly to assist with long‐range signaling (Coombs et al. 2010). Thus, lipid modifications play a crucial role in regulating Wnts.

#### 2.2.4 Shh Signaling

Shh, synthesized as a 45 kDa precursor protein, undergoes N‐terminal signal peptide cleavage, then C‐terminal auto‐processing that incorporates a cholesterol modification, as well as N‐terminal cysteine palmitoylation via an amide linkage, to form a 19 kDa mature signaling molecule. Palmitoylation is important for long‐range and short‐range signaling. This is catalyzed by Hedhehog acyltransferase, a palmitoyl transferase (Buglino and Resh 2012). Shh signaling plays an important role in mammalian body patterning, including the development of the neural tube and ventral forebrain structures (Echelard et al. 1993; Ericson et al. 1995). Shh in the developing cortex regulates the proliferation of RGs and BPs, survival, differentiation, lamination, and specification of neurons (Komada 2012; Araújo et al. 2014; Wang et al. 2016; Oishi et al. 2022). Studies in Drosophila identified that the expression of Shh that lacks the cholesterol moiety led to an extended range but less effective signaling, and palmitoylation of Shh is critical for its activity (Dawber et al. 2005). Reports suggest that N‐terminal palmitoylation of cysteine is differentially required in Drosophila compared to mouse, with wing patterning activity in Drosophila being more affected versus unaffected limb patterning in mouse upon deacylation of Shh (Lee et al. 2001). However, the fatty acylation of Shh is found to potentiate its ability to differentiate ventral forebrain neurons, and injection of embryonic forebrain with the unacylated form of Shh failed to recapitulate the effects of wildtype‐Shh (Kohtz et al. 2001). Therefore, it cannot be neglected that the effects and requirements could be tissue‐specific. Studies also suggest that both cholesterol addition and palmitoylation of Shh in mouse are important for formation of Shh multimeric protein complexes and long‐range signaling (Lewis et al. 2001; Chen et al. 2004).

### 2.3 Fatty Acid Synthesis Regulating Embryonic NSCs

**FIGURE 3:**
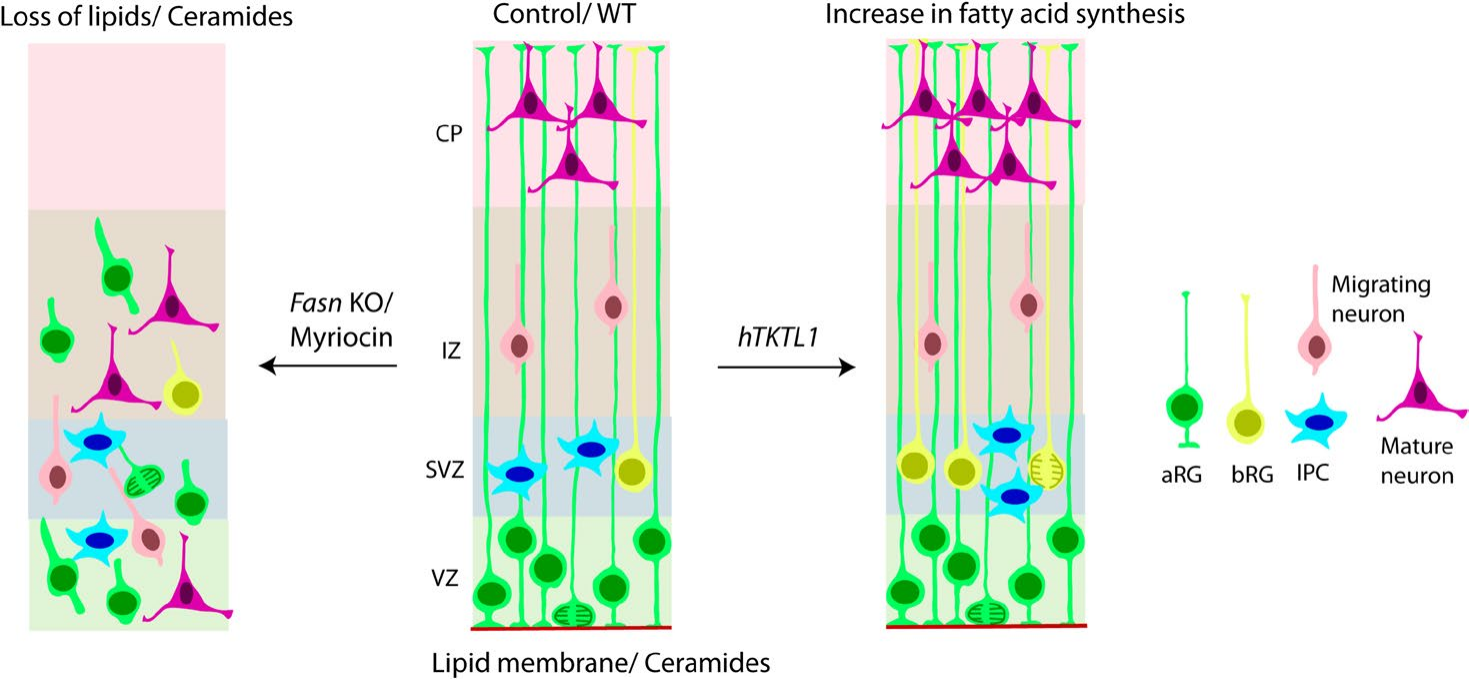
Importance of lipids in cerebral cortex development. Lipids/ceramides are enriched in plasma membranes of cortical cells. Reduced generation of palmitate and subsequent lipids upon knockout (KO) of fatty acid synthase (*Fasn*), or ceramides upon treatment with myriocin, leads to loss of polarity and a disorganized cortex. Expression of a modern human variant of transketolase 1 (*hTKTL1*) in mouse leads to an increased production of bRGs and consequently an increased number of upper layer neurons upon increased fatty acid synthesis. aRG, Apical radial glia; bRG, Basal radial glia; IPC, Intermediate progenitor cell. Data from (Gonzalez‐Bohorquez et al. 2022; Pinson et al. 2022; Wang et al. 2008).

It has already been shown that *de novo* lipogenesis is crucial for proper cortex development. Conditional inactivation of *Fasn*, the gene encoding fatty acid synthase, a primary enzyme of lipid biogenesis, in the mouse embryonic forebrain, led to disorganization of cortical layers upon loss of apico‐basal polarity of RGs (Figure 3), causing reduced cell proliferation and severe microcephaly. Deletion or pharmacological inhibition of *FASN* in human forebrain organoids also led to polarity defects affecting the RG scaffold, confirming the conservation of function in humans (Gonzalez‐Bohorquez et al. 2022). Thus, fatty acid synthesis is crucial for RG structure and proliferation.

Studies by Pinson and colleagues identified that the expression of the modern human variant of transketolase 1 (hTKTL1, involved in lysine‐to‐arginine amino acid substitution), but not the Neanderthal variant, increased the abundance of bRGs in mouse and ferret animal models. *hTKTL1* gene modification to introduce the Neanderthal variant in fetal human neocortical tissue and cerebral organoids lowered the number of bRGs, illustrating the specific importance of the human variant. It is interesting that the increased bRG abundance (Figure 3) was achieved through two metabolic pathways of the pentose phosphate pathway (PPP) followed by fatty acid synthesis. TKTL1 is reported to cleave Xylulose 5‐phosphate into glyceraldehyde 3‐phosphate and acetate in the PPP. Glyceraldehyde 3‐phosphate converted to pyruvate in the glycolysis process will then produce acetyl‐CoA, a critical metabolite for fatty acid synthesis (Figure 1). Acetyl‐CoA can also be processed from the acetate produced in the PPP. Expression of hTKTL1 therefore led to an increased concentration of acetyl‐CoA, expected to favor fatty acid synthesis. Inhibition of either the PPP or fatty acid synthesis reduced hTKTL1‐induced bRG abundance, and it was proposed that hTKTL1 promotes the synthesis of membrane lipids necessary for the outgrowth of bRG processes and hence for their increased abundance (Pinson et al. 2022). These data illustrate the importance of fatty acids in neocortical expansion during evolution.

## 3 Ceramides and Sphingolipids in Brain Development

Fatty acids are used to generate ceramides, which then can form more complex sphingolipids in the Golgi apparatus. Perturbation of ceramide synthesis pathways leads to neurodevelopmental defects mentioned here.

### 3.1 Neural Tube Formation

Maternal exposure to corn crops contaminated with fungal mycotoxins, for example, fumonisins, can lead to an increased incidence of neural tube defects in infants in populations where corn consumption is higher (Marasas et al. 2004; Missmer et al. 2006). Fumonisin is known to inhibit ceramide synthases and lead to an accumulation of intermediates of sphingolipid metabolism and/or depletion of complex sphingolipids. Fumonisin treatment of Caco‐2 cells led to a significant reduction in receptor‐mediated folate uptake (Stevens and Tang 1997). The placental high‐affinity folate transfer protein is a glycosylphosphatidylinositol (GPI)‐anchored protein that associates with membrane rafts enriched in cholesterol and sphingolipids (Luhrs and Slomiany 1989; Brown and London 1998). Treatment with Fumonisin was found to alter the endocytic trafficking of the transporter and also the amount of receptor available for transport (Stevens and Tang 1997; Chatterjee et al. 2001). Exposure to Fumonisin caused neural tube defects in mouse embryos in culture and in vivo. Supplementation with folate or folic acid partially reduced the incidence of these defects (Sadler et al. 2002; Gelineau‐Van Waes et al. 2005). GPI‐anchored receptors, including the folate receptor, are known to associate with gangliosides, and pregnant mice administered with ganglioside GM1 alongside fumonisin treatment restored folate levels and majorly reduced the incidence of neural tube defects (Gelineau‐Van Waes et al. 2005). This clearly illustrates the importance of sphingolipids for folate receptor attachment and subsequent functioning. Studies by Callihan and colleagues proposed that the accumulation of intermediates dihydrosphingosine and dihydrosphingosine‐1‐phosphate (dhS1P) could lead to the neurodevelopmental defects observed in the above‐mentioned cases, and they showed experimentally that dhS1P has more potent activity at S1P receptors in human NSCs (Callihan et al. 2012).

### 3.2 Early Brain Development

Targeted depletion of sphingolipids by inhibition of *de novo* ceramide biogenesis in vitro in embryonic stem (ES) cell‐derived neural progenitors, using inhibitors such as Myriocin or Fumonisin B1, led to their reduced motility, and the effect was rescued upon addition of endogenous ceramide species or analogues, but notably, not short‐chain ceramide species.

Further incubation of neural progenitors with anti‐ceramide antibodies reduced the formation of membrane protrusions and lamellipodia, resulting in rounded‐up cells forming clusters. Thus, ceramide is important for cell morphology and motility. Interestingly, this phenotype was accompanied by the translocation of membrane‐bound atypical protein kinase C (aPKC, a polarity protein) to the cytosol and nucleus. It was previously known that ceramide binds to aPKC and activates it (Wang et al. 2005). Ceramide co‐distributes with aPKC, Cdc42, α‐tubulin, and β‐catenin, and treatment with anti‐ceramide antibody prevented the membrane translocation of Cdc42 and co‐distribution with aPKC and β‐catenin. It was proposed that the ceramide‐mediated membrane translocation of aPKC and Cdc42 inhibits GSK‐3β, and this in turn stabilizes β‐catenin and microtubules, aiding NSC polarity and motility (Wang et al. 2008). In vivo depletion of sphingolipids in mouse embryonic telencephalon by myriocin treatment depleted the accumulation of ceramide in the apical membrane along with the apical co‐distribution of aPKC and β‐catenin, leading to the disorganization of cortical layers, with an ectopic localization of immature neurons in the germinal zones (VZ, SVZ) (Wang et al. 2008). This phenotype was rescued upon the addition of the ceramide analogue S18 (Wang et al. 2008). This illustrates the importance of ceramides and sphingolipids, particularly in cell (NSC) polarity during cortex development.

The differential expression of gangliosides (complex sphingolipids) during NSC proliferation and differentiation, axogenesis and dendritic arborisation, synaptogenesis and myelination emphasizes their importance at different stages of brain development. The simple gangliosides such as GM3 and GD3 are found to predominate at the earlier stages of embryonic development and the more complex gangliosides such as GM1, GD1a, GD1b, and GT1b are present at later stages (Ngamukote et al. 2007; Olsen and Færgeman 2017). The ganglioside GD3 is detected at high levels in the NSCs of embryonic mouse brains. Analyses of GD3 KO‐derived NSCs revealed decreased self‐renewal capacity with reduced EGFR expression and EGF‐induced ERK signaling. It further revealed defects in the endocytic pathway with more EGFR in GD3 KO‐NSCs passing through the endosomal‐lysosomal degradative pathway rather than the recycling pathway (Wang and Yu 2013). Further studies in postnatal mouse brains also confirmed the crucial role of the ganglioside GD3 in the self‐renewal capacity and maintenance of adult NSCs in the SVZ and the dentate gyrus of the hippocampus (Wang et al. 2014).

The expression of gangliosides GM1 and GM3 was also detected in proliferative RGs in the VZ of the early developing human cerebral cortex between gestational weeks 6 and 15. In addition, GM1 was more intense at membrane contact sites in the membrane of neurons migrating along RG fibers, suggesting a role in radial migration (Stojiljković et al. 1996). The distribution of an acetylated form of ganglioside GD3, 9‐O‐acetyl GD3, was found to correlate both spatially and temporally with neuronal migration and neurite outgrowth in different regions in the central and peripheral nervous system of the rat, including their radial organization associated with radial migration in the developing telencephalon (Mendez‐Otero et al. 1988; Schlosshauer et al. 1988; Mendez‐Otero and Santiago 2003). Thus, gangliosides play crucial roles during development.

### 3.3 Axon and Dendritic Growth

Primary studies found that the addition of gangliosides can promote neurite outgrowth (Roisen et al. 1981; Ferreira et al. 1990). Also, inhibition of the ceramide biogenesis enzyme, ceramide synthase, has shown defects in axonal growth and dendritic branching in several studies. Firstly, inhibition of ceramide synthesis by Fumonisin B1 in cultured hippocampal neurons led to defects in axon growth owing to a reduction in ganglioside synthesis, which possibly supplies new membrane material to the growing axon (Harel and Futerman 1993). Following studies which used an inhibitor of ceramide synthesis or glucosylceramide synthesis led to defects in the length of the axon plexus and branching, rescued by inhibition of glucosylceramide degradation, confirming the involvement of gangliosides in this process (Schwarz et al. 1995). Further studies which examined the effects of inhibition of ceramide biogenesis in developing rat cerebellar Purkinje neurons in vitro found decreased cell survival, as well as alterations in dendritic morphology and branching upon reduction of certain gangliosides, such as GD1a, 9‐O‐acetylated LD1, GD3, and sphingomyelin (Furuya et al. 1995, 1998). Interestingly, the addition of cell permeable ceramide or its derivatives rescued these abnormalities (Furuya et al. 1998).

Studies of sphingolipid biogenesis enzyme knockouts illustrate their increased importance at later stages of development including axon development and myelination. Glucosylceramide synthesis is primarily catalyzed by the enzyme UDP‐Glucose ceramide glucosyltransferase (Ugcg), more commonly known as glucosylceramide synthase (GCS). Global *Ugcg* KO mice were embryonic lethal (Yamashita et al. 1999). A Nestin‐Cre conditional KO of *Ugcg* was generated to specifically study effects in neural cells: the KO mice developed severe ataxia and displayed neuronal defects at postnatal stages, although not likely impacting embryonic development, when judged based on gross macroscopic observations until birth. These mice died 3 weeks after birth with observations of structural defects affecting the cerebellum and peripheral nerves, including reduced axonal branching of Purkinje cells, as well as broadened and severely disorganized myelin sheaths of peripheral nerves. The defects also included diminished dendritic complexity of the cultured hippocampal neurons accompanied by early pruning. Interestingly, the impacted processes involved pathways important for brain development and homeostasis including Mapk, Wnt, Cxcr4, and G protein‐coupled receptor signaling (Jennemann et al. 2005). Purkinje cell‐specific *Ugcg* KO mice exhibited swollen axons with axonal degeneration and myelin sheath defects with detached paranodal loops and double myelination of axons (S. Watanabe et al. 2010). Another mouse line devoid of all ganglio‐series of glycosphingolipids developed weakness of the hind limbs, ataxia, and tremors at 2 weeks of age and death in the majority of cases, a few weeks following weaning at 3 weeks. The morphological phenotypes include decreased brain size at 1 month of age followed by severe neurodegeneration between 2 and 3 months with prominent vacuolization, and accumulation of astrocytes and oligodendrocytes with massive cytoplasmic vacuoles in the white matter regions, with abnormal paranodal junctions at the nodes of Ranvier indicating possible impairment of axon‐glial interactions (Yamashita et al. 2005). Mice just devoid of complex gangliosides survived to adult stages and displayed nervous system defects, sometimes mild, while particularly affecting the later stages of development including myelination and general homeostasis in accordance with their expression pattern suggesting their functional relevance (Takamiya et al. 1996; Sheikh et al. 1999; Kawai et al. 2001; Inoue et al. 2002; Sugiura et al. 2005). The defects included nerve degeneration, susceptibility to lethal seizures, decreased myelination, axonal degeneration, and demyelination (Sheikh et al. 1999; Kawai et al. 2001; Inoue et al. 2002; Sugiura et al. 2005).

Analyses of two mouse models for cerebellar ataxia and Purkinje cell degeneration with spontaneous recessive mutation in the *ceramide synthase 1* (*Cers1*) gene (*Flincher* (*fln*) and *toppler* (*to*)) showed a complete loss of Cers1 activity and a reduction in C_18_ sphingolipid biogenesis with increased levels of C_14_ and C_16_ sphingolipids, as well as common substrates such as sphingosine, dihydrosphingosine, and their phosphorylated metabolites. Ceramide profile changes have also been previously reported in certain neuronal ceroid lipofuscinoses (CLNs) patients. Similarly, when *Cers1* mouse mutants *fln* and *to* were screened, accumulation of lipofuscin with ubiquitylated proteins was found in different brain regions including deep cerebellar nuclei, pons, medulla, anterior olfactory nuclei, cerebral cortex, and hippocampus (Zhao et al. 2011). Mutations in *CERS1* and *CERS2* have been associated with progressive myoclonic epilepsy in humans (Mosbech et al. 2014; Vanni et al. 2014; Afonso et al. 2015; Godeiro Junior et al. 2018), and analyses of *Cers1/2* KOs revealed neurodegeneration and/or myelin sheath defects (Imgrund et al. 2009; Ginkel et al. 2012). Interestingly, *Cers1* deficiency induced neuronal death upon accumulation of long‐chain base substrates utilized by all Cers isoforms such as dihydrosphingosine, sphingosine, their phosphorylated metabolites, and 1‐deoxysphinganine, and this was rescued upon ectopic expression of Cers2. This illustrates that the phenotype observed here is primarily caused by the unutilized toxic substrates rather than the defective production of specific ceramide/sphingolipid species (Spassieva et al. 2016).

### 3.4 Myelination

Myelin, an extension of the plasma membrane of oligodendrocytes in the central nervous system (CNS) and of Schwann cells in the peripheral NS (PNS), is composed of lipids (> 70% of the dry weight) and myelin‐specific proteins (Coetzee et al. 1996; Quarles et al. 2006). Galactocerebroside and its sulphated derivative, sulfatide, together represent ∼27% of myelin lipid (Norton and Cammer 1984; Coetzee et al. 1996). Inhibition of galactocerebroside and sulfatide synthesis in mice lacking UDP‐galactose:ceramide galactosyltransferase (CGT) did not affect myelin structure, although slightly thinner sheaths were observed in the ventral region of the spinal cord. However, the mice also suffered generalized tremor and mild ataxia with conduction deficits upon reduced insulative capacity of the myelin sheath (Bosio et al. 1996; Coetzee et al. 1996). With age, they also developed progressive hindlimb paralysis and extensive vacuolation of the ventral spinal cord (Coetzee et al. 1996; Bosio et al. 1998). Subsequent analyses of the CGT mutant mice using electron microscopy revealed several nodal and paranodal defects in the CNS, suggesting that myelin galactolipids are important for axo‐oligodendrocytic interactions that ensure proper nodal and paranodal formation (Bosio et al. 1998; Dupree et al. 1998). A paranodal protein, Paranodin, which is a neuronal mediator of axon‐myelin binding, was shown to be diffusely distributed along the internodal regions rather than being concentrated in the paranodal regions (Dupree et al. 1999). Inhibition of sulfatide synthesis alone by deleting the cerebroside sulfotransferase gene (CST) in mice also led to paranodal junction abnormalities (Honke et al. 2002). Mice that are devoid of the myelin‐associated glycoprotein (MAG) and galactolipids, both important for mediating axo‐glial interactions during the myelination process, displayed more severe abnormalities at the nodes of Ranvier than single mutants, suggesting the overlapping function of both (Marcus et al. 2002).

### 3.5 Synapse Formation

Lipid rafts consisting of cholesterol/sphingolipid microdomains are found to be abundantly present in dendrites of cultured hippocampal neurons where they are associated with postsynaptic proteins including AMPA receptors. Drug‐induced depletion of cholesterol/sphingolipid led to an increased internalization of AMPA receptors and subsequently loss of synapses and dendritic spines (Hering et al. 2003). Thus, sphingolipids are implicated also in synapse formation. For more detailed information regarding the role of sphingolipids in synapse formation and plasticity, we refer readers to (Olsen and Færgeman 2017; Hussain et al. 2019).

## 4 Ceramides in Cell Compartments

### 4.1 Ceramides in Primary Cilia

**FIGURE 4:**
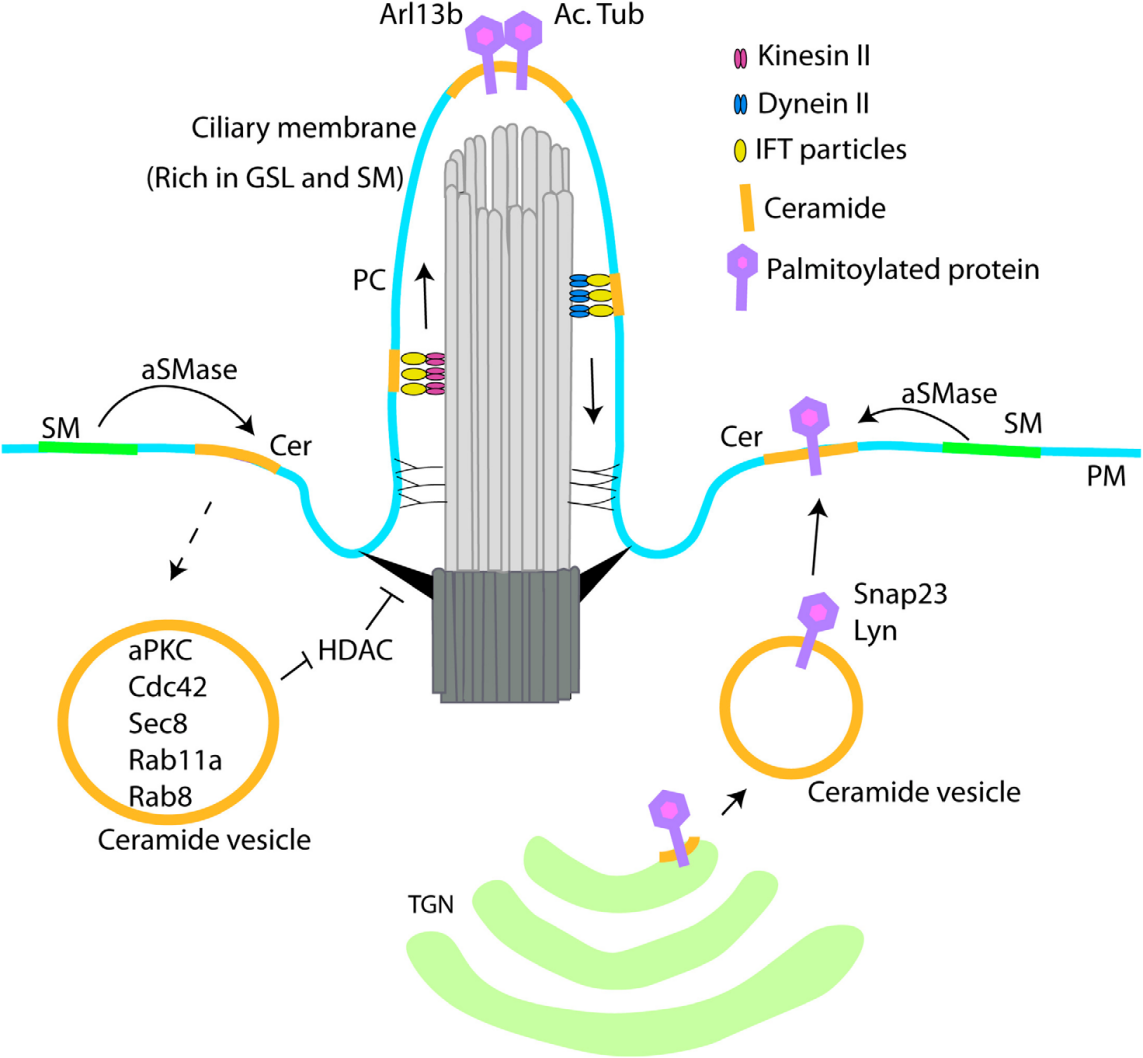
Importance of ceramides in vesicular trafficking of proteins to the plasma membrane and formation of primary cilia. Acid sphingomyelinase (aSMase) derived ceramides assist with the vesicular transport of palmitoylated proteins from the Golgi apparatus to the plasma membrane. SMase‐derived ceramide colocalizes with Rab11a vesicles containing polarity and ciliogenic complex proteins, and promotes acetylation of tubulin in cilia by inhibiting HDAC. Ceramide in primary cilia membranes mediates membrane anchoring and interaction between palmitoylated proteins. Ceramide also maintains the structure of primary cilia by interacting with IFT particles that connect the membrane to the axoneme. aSMase, acid sphingomyelinase or secreted acid sphingomyelinase; Cer, Ceramide; PC, Primary cilia; PM, Plasma membrane; SM, Sphingomyelin. Data from (He et al. 2012; Tripathi et al. 2021; Wu et al. 2022; Xiong et al. 2019).

The primary cilium, a sensory organelle, is a microtubule‐based structure, important during the cell cycle, including in RGs (Uzquiano et al. 2019; Zaidi et al. 2022). Gangliosides GM₁ and GM₃ were detected in the primary cilia of polarized epithelial cells (Janich and Corbeil 2007). In polarized Madin‐Darby Canine Kidney (MDCK) cells, the sphingolipid ceramide was found to be specifically distributed to a cis‐Golgi compartment at the base of the primary cilium and to cause inhibition of GSK‐3β (G. Wang et al. 2009). Furthermore, the ceramide present in an apical ceramide‐enriched compartment was derived from apical sphingomyelin upon the action of the enzyme acid SMase (Figure 4). Apical ceramide colocalizes with Rab11a vesicles containing polarity and ciliogenic complex proteins such as aPKC, Cdc42, Sec8, and Rab8, and independently promotes acetylation of tubulin in cilia (He et al. 2012). The regulation of ciliogenesis by ceramides was found to be conserved in human embryonic stem cell‐derived neuroprogenitors and further, they were found to prevent activation of the deacetylase HDAC6 by cytosolic aPKC (normally tethered to the membrane by ceramide) and AurA, thereby promoting acetylation of tubulin in primary cilia (He et al. 2014).

Translatome profiling during cilia regeneration in *Chlamydomonas* identified sphingolipid metabolism enzymes including the rate‐limiting enzyme of sphingolipid biogenesis, serine palmitoyltransferase (SPT), as important regulators for ciliogenesis. Further cryo‐electron tomography studies revealed that loss of ceramide abnormally increased the membrane–axoneme distance and led to the formation of swollen cilia. It was found that ceramides interact with the intraflagellar transport (IFT) particle proteins, of which IFT motors perform transport along axoneme microtubules, indicating a connection between the ciliary membrane and axoneme to form a normal rod‐shaped cilium. Further studies in mouse IMCD3 cells and zebrafish lateral line neuromasts and olfactory placode identified the ciliary localization of ceramides and conservation of function in cilium formation in these cells (D. Wu et al. 2022).

Lipids have also been implicated in controlling signaling at the primary cilia. A CRISPR screen of lipid‐related genes identified cholesterol biogenesis enzymes as positive regulators, and sphingolipid biogenesis enzymes as negative regulators of Shh signaling (Kinnebrew et al. 2019). Depletion of sphingomyelin by myriocin or fumonisin B1 treatment in NIH/3 T3 cells potentiated the Shh signaling response, and it was reversed upon exogenous addition of sphingomyelin (Kinnebrew et al. 2019). The effect was found to be conserved in two other cell types: mouse embryonic fibroblasts and mouse spinal NSCs. Depletion of sphingomyelin, which normally sequesters cholesterol in the ciliary membrane, increases cholesterol accessibility and amplifies Shh signaling (Kinnebrew et al. 2019).

As mentioned, primary cilia are microtubule‐filled, antenna‐like structures which in RGs in interphase, protrude in the cerebral ventricle, receiving signals from the CSF (Zaidi et al. 2022). *ARL13b*, a gene implicated in Joubert syndrome and several neurodevelopmental defects, undergoes palmitoylation which regulates its stability and trafficking into the primary cilia (Cevik et al. 2010; Roy et al. 2017). Indeed, a ceramide‐rich platform in cilia has been identified to mediate membrane anchoring and interaction between two palmitoylated proteins, acetylated tubulin and Arl13b (Tripathi et al. 2021). Another study using mass spectrometry‐based proteomic analyses identified proteins associated with acid SMase‐dependent lipid rafts, the majority of which are palmitoylated proteins including Snap23, Yes, Lyn, Ras, and Rab family small GTPases. Thus, acid SMase‐derived ceramides may be important for vesicular transport of palmitoylated proteins (Snap23 and Lyn) from the Golgi apparatus to the plasma membrane (Xiong et al. 2019). Although not many studies have been performed to investigate the interaction of lipid‐modified proteins with ceramides, these few studies illustrate the importance of ceramides in the trafficking, protein–protein interaction, and membrane interaction of palmitoylated proteins.

### 4.2 Ceramides in Vesicles in Polarized Cells

Ceramides have been clearly demonstrated to be involved in the formation of extracellular vesicles (Trajkovic et al. 2008; Menck et al. 2017). Exosomes derived from the intraluminal vesicles of multivesicular endosomes of Oli‐neu cells (a mouse oligodendroglial cell line) were enriched in cholesterol and sphingolipids. Importantly, there was an enrichment of ceramides in exosomes, and these were generated in an ESCRT‐independent manner by ceramide‐dependent budding of intraluminal vesicles. Exosome release was found to be greatly reduced after treatment with neutral SMase inhibitors, suggesting the importance of ceramides in this process (Trajkovic et al. 2008, but see below). Treatment of neurons with these inhibitors also decreased the release of exosomes, and inhibition of sphingomyelin synthase enhanced the release of exosomes. In polarized epithelial cells, exosome release from the apical side was found to be dependent on ALIX‐Syntenin1‐Syndecan1 machinery, and basolateral release used SMase‐dependent ceramide production machinery (Matsui et al. 2021). However, neutral SMase inhibition may affect the secretion of microvesicles in different ways, in some cases causing increased secretion and affecting the balance of extracellular vesicle release (Menck et al. 2017). In glial cells, activation of acid SMase through the p38 MAPK cascade was associated with an increase in microvesicle release, with its blockade leading to a decrease (Bianco et al. 2009). RGs being highly polarized, this type of regulation could greatly influence their function. Extracellular vesicles from different neural cells, including NSCs, were found to carry several important neurodevelopmental factors and influence the cell fate of the recipient cells (Kyrousi et al. 2021; Pipicelli et al. 2023; Forero et al. 2024). It will be interesting to decipher how ceramides impact extracellular vesicle release from these cells.

Apart from extracellular vesicles, intracellular trafficking of proteins also depends on ceramides (Pepperl et al. 2013; Xiong et al. 2019). Sterols and sphingolipids were found to be enriched in the Golgi‐derived apical transport carrier membranes in yeast (Klemm et al. 2009). Studies by Duran and colleagues manipulated the sphingomyelin levels of the Golgi membrane by exogenous addition of D‐ceramide‐C6 to HeLa cells and found that C6‐sphingomyelin reduced the lipid order in the Golgi membranes, leading to inhibition of transport carrier formation (Duran et al. 2012). Downregulation of Drosophila's only ceramide synthase gene, *schlank*, in wing disc led to impaired endosomal trafficking of proteins. One of the affected and studied proteins, Wg, although present at higher levels in vesicles, showed overall reduced signaling activity. This effect was found to possibly be due to defective formation of Rab7 late endosome and lysosome structures, suggesting a defective early‐to‐late endosomal trafficking route upon ceramide depletion (Pepperl et al. 2013). Ceramides have also been found to be crucial for post‐Golgi trafficking of palmitoylated proteins, such as Snap23 and Lyn, to the plasma membrane (as described above) (Xiong et al. 2019). Ceramide is enriched in Rab11a vesicles along with other ciliogenic proteins at the base of the primary cilia in MDCK cells, and the inhibition of acid SMase disrupted the association of ciliogenic proteins with these vesicles, affecting cilium formation. C16 and C18 ceramides, although less abundant, were increasingly found in the ceramide‐containing Rab11a vesicles, suggesting the necessity to decipher the functional relevance of different ceramide species (He et al. 2012).

### 4.3 Ceramides and Mitochondrial Function

Mitochondria are key organelles during neurodevelopment; their regulation, dynamics, and localisation have been shown to be important during several steps of corticogenesis. As well as energy production to meet the special needs of the different cell types, mitochondria also take part in the production of second messengers to induce cellular changes and fate acquisition. These changes may originate from lipid regulation, of which comparatively little is known concerning sphingolipid and ceramide relevance in mitochondria during neurodevelopment, despite the fact that information concerning sphingolipids during neuronal processes such as aging has been explored; hence, more studies are needed to investigate this.

During lifespan, ceramide content in the brain fluctuates to meet the requirements of cell function. During the postnatal period in rats, sphingolipid levels fluctuate in brain mitochondria, and this may be related to a second wave of neuron elimination (Novgorodov et al. 2011). Brain levels of sphingomyelin, sphingosine, and most ceramide species increase with time, but levels of ceramides C16:0 and Cers6 decrease. This downregulation is associated with a reduced mitochondrial Ca^2+^ loading capacity, one process which can promote apoptosis. This mechanism was observed and described in oligodendrocytes (Novgorodov et al. 2011).

Mechanistically, how ceramides and CERSs impact mitochondrial function and dynamics has been poorly described in neuronal cells, but is extensively studied in cancer cells (Alizadeh et al. 2023). Ceramides can self‐assemble to form ceramide channels in mitochondrial outer membranes (MOM) (Colombini 2019). Also, a synergistic effect was observed between ceramides and Bcl_2_ family proteins, which are known to be involved in MOM permeabilization. The interaction between ceramides and Bax and the ability of ceramides to form channels in the MOM suggests multiple mechanisms allowing cytochrome C release in the cell and leading to apoptosis. This information highlights multiple levels of ceramide activity. Ceramides can also act as second messengers and activate VDAC2 or inhibit BCL2 and regulate Bax. They act at different levels of the apoptotic pathway. Interestingly, different ceramide species and CERS family members can have either anti‐ or pro‐apoptotic effects in various cell types (Patwardhan et al. 2016; Ogretmen 2017; Alizadeh et al. 2023). Therefore, studying these mechanisms in the context of neuronal cell types during brain development is important to fully decipher the importance of their differential distribution and expression on mitochondrial dynamics and function.

Ceramides and associated enzymes are hence differently present in mitochondria during brain development, leading to different cellular responses, but these mechanisms may also be linked to cellular identity. Cholesterol is known to promote the survival of newly formed neurons; this mechanism is hypothesized to be due to a differential repartition of cholesterol interactors PAR4 and ceramides in daughter cells after progenitor asymmetrical division (Bieberich et al. 2003). Authors showed that the reduction of ceramides reduces progenitor apoptosis, whereas treatment with ceramide analogues elevated apoptosis. Furthermore, they observed an asymmetrical distribution of Nestin and PAR4 during the production of newly born neurons. Daughter cells which were Nestin negative and PAR4 positive presented elevated ceramide levels and apoptosis, whereas Nestin positive and PAR4 negative daughter cells did not display this increase and did not undergo apoptosis. The differential distribution of ceramides during asymmetrical division could hence be linked to the cellular identity of newly born neurons.

Another important role of mitochondria during neurogenesis was shown by Iwata et al., with differential mitochondrial dynamics occurring during neurogenesis. After progenitor division, the daughter cell that will self‐renew undergoes mitochondrial fusion, and the newly born neurons present mitochondrial fission (Iwata et al. 2020). In line with this work, ceramides are known to be involved in such dynamics in hypothalamic neurons after diet‐induced morphological alterations (Hammerschmidt et al. 2023). A high‐fat diet induces mitochondrial fragmentation in liver cells, and full KO of the *Cers6* gene reduces the observed effect (Hammerschmidt et al. 2019). This was also observed in hypothalamic neurons in culture; Cers6 and ceramide C16:0 depletion rescued the mitochondrial fragmentation. Of note, in normal conditions, in hypothalamic neurons deficient in Cers6, no changes in mitochondria dynamics were observed (Hammerschmidt et al. 2023). Therefore, it will be interesting to study the impact of Cers6 and C16:0 ceramides on mitochondria morphology during corticogenesis to see if the observation remains true in progenitor cells undergoing asymmetrical division and playing a potential role in neurogenesis.

### 4.4 Ceramides and Their Derivatives as Signaling Molecules Influencing Cell Survival

Sphingolipids have been implicated in various cellular functions, such as cell growth, cell cycle, cell death, senescence, adhesion, migration, response to stress, and autophagy. These processes have been studied in different cell types (Hannun and Obeid 2018). Some have been described in a neuronal context but without a focus on brain development. Depending on the ceramide doses, they can trigger or protect from neuronal apoptosis (Colombaioni and Garcia‐Gil 2004). Therefore, it is important to study in a specific manner the impact of different ceramides in specific neuronal cell types at various time points of neurodevelopment. An example of this effect was shown by Riebeling et al., as they tried to decipher the differential impact of ceramides from the *de novo* and salvage pathways on hippocampal neuronal growth. They showed that the production of ceramides from sphingomyelin at the membrane at earlier stages enhances the production of minor neuritic processes from lamellipodia, later on defined as axons. At later stages, glycosylated ceramide is also implicated in axonal growth. These two processes are tuned by neurotrophic pathways and are limited by the amount of ceramides, an excess leading to neuronal apoptosis (Riebeling and Futerman 2002).

Another well‐described mechanism in cancer cells related to ceramide is the response to ER stress. Ceramides are produced in the ER, and many enzymes related to sphingolipid synthesis are present in the ER (Figure 1). Therefore, their modulation is tightly linked to ER homeostasis. In cancer cells, the species of ceramides (acyl chain length) will have differential effects on the response to ER stress. Several sphingolipid metabolites act at different areas of the ER stress response with opposite effects. Therefore, it is important to characterize them in a cell‐specific manner. For example, downregulation of Cers6 and decreased ceramide C16:0 will trigger BiP/ATF‐6 activation, Ca2+ release from the ER, and Golgi membrane fragmentation, disrupting the ER‐Golgi network and activating the unfolded protein response (UPR) pathway. Moreover, Cers2 and very long‐chain ceramides will inhibit PERK and ATF6 activation (Park and Park 2020). Along this line, Contreras et al. were able to show in a model of obesity that hypothalamic neurons in vivo, after being stimulated with ceramide analogues, responded with increased ceramides (C16) in the hypothalamus and ER stress with the activation of the UPR pathway (Contreras et al. 2014). Of note, these studies often show stimulated ceramide production with exogenous ceramide analogues; this approach is non‐specific to one species of ceramide and associated enzymes, making causal conclusions difficult. To bypass this problem, using tools to downregulate one enzyme of the pathway is helpful to study specific ceramide species. Spassieva et al., using a neuroblastoma cell line and siRNA, downregulated Cers2. They showed an accumulation of long‐chain ceramides, triggering cell cycle arrest and induction of autophagy via the UPR pathway, but no apoptosis (Spassieva et al. 2016). These mechanisms have not yet been described during neurodevelopment in specific cell types but need to be investigated.

Lastly, ceramides are important second messenger transducers related to the formation of lipid rafts at membranes. In the brain, lipid rafts differ in their composition in a cell‐specific manner. In neurons, lipid rafts are depleted in phospholipids and enriched in sphingomyelin, cholesterol, ceramides, and glycosphingolipids (Colombaioni and Garcia‐Gil 2004). This sphingolipid composition in rafts also depends on brain age, as more ceramides are found in aging neurons (Prinetti et al. 2001). The presence of rafts enriched in ceramides is hypothesized to be important for the presence of larger platforms allowing receptor clustering and oligomerization (Colombaioni and Garcia‐Gil 2004). In cancer cells, increased ceramide levels will enhance CD95 clustering at the membrane, facilitating trimerization and activation. This mechanism will activate PERK signaling and the ER stress response (Park and Park 2020). A similar effect was observed in oligodendrocytes related to myelination; dihydrosphingomyelin at the membrane promotes the formation of dhCer microdomains, increasing membrane rigidity. This effect is linked to increased cell death, cell cycle arrest with autophagy, and ER stress modulation (Tzou et al. 2023).

In the same line as ER stress, autophagy is linked to ceramide regulation. Different ceramide species trigger different outcomes in cellular responses in cancer cells. Jiang et al. refer to this as the “autophagy paradox”, as they can either be protective or deleterious for cell survival. Several pathways have been described in depth in cancer cells (Jiang and Ogretmen 2014). However, not much is known in neuronal cells, often activating ER stress in relationship with autophagic responses, as mentioned previously.

### 4.5 Membrane Rigidity

Sphingolipids and ceramides act as building blocks of cell membranes, and their differential distribution in cell compartments allows local changes in membrane physicochemical properties. Sphingolipids display a long and saturated fatty acyl chain; these lipid properties make membranes thicker and less fluid. These domains are liquid‐ordered and allow a strong lipid‐lipid interaction. These nanodomains are hubs for transmembrane proteins and are stabilized by cholesterol and sphingolipid (Harayama and Riezman 2018). However, they also influence overall plasma membrane fluidity and cell motility. In cancer cells, epithelial to mesenchymal transition (EMT) is an important step in metastatic events, reflecting cell motility. EMT‐like events are also observed during neurodevelopment as cells leave the neuroepithelium. It was shown that Cers6 expression was decreased during EMT, influencing ceramide C16 levels and plasma membrane fluidity. Therefore, cancer cells display increased motility and infiltration (Edmond et al. 2015). This effect is of interest in the case of neurodevelopment as progenitor cells undergo many morphological changes and neurons undergo migratory events. It would be of interest to understand if ceramide level modulation could impact membrane properties during these highly dynamic events. These changes in sphingolipid and ceramide composition are aided by lipid transport proteins found at different subcellular localisations (Chiapparino et al. 2016), as well as the properties of these lipids. Ceramides can perform rapid flip‐flop movement across cell membranes, promoting a trans‐layer movement of other lipids (Contreras et al. 2003; López‐Montero et al. 2005). This specific subcellular localisation and dynamic also allows the formation of ceramide pores. These pores are found in organelles, such as mitochondria, acting on mitochondrial permeabilization and function (Colombini 2019).

Overall, sphingolipids and ceramides are crucial for cell functions, acting as bioactive lipids, second messengers, and building blocks of cellular membranes. Their diversity and redundancy allow for tight regulation of cellular functions in a cell type and time‐regulated manner. These mechanisms have not yet been well studied in the brain, including during neurodevelopment; therefore, characterizing them is crucial to decipher their roles.

## 5 Ceramides and Sphingolipids in Neurological Disorders

The importance of studying sphingolipids and their associated enzymes helps to shed light on their relevance with respect to different neuropathological and neurodevelopmental disorders. In the last decades, increasing evidence and pathological cases have linked the importance of lipid homeostasis in the nervous system and how their imbalances can lead to severe neuropathologies, ranging from neurodegenerative disorders including Alzheimer disease (AD), Parkinson disease (PD), and leukodystrophy to epilepsy, intellectual disabilities (ID), Joubert Syndrome (as mentioned above), and neuropsychiatric disorders.

Most patients display multifactorial pathologies, highlighting the importance of sphingolipid homeostasis in different cell types and in a developmental manner. This homeostasis can be perturbed at different levels, triggered by mutation in specific enzymes of the sphingolipid pathway or by changes in sphingolipid composition due to cell responses. Some cases are intricate since patients appear to display both neurodevelopmental and neurodegenerative phenotypes.

### 5.1 Neurodegenerative and Neurometabolic Disorders

Studies and reviews have linked several species of lipids and enzymes to neurodegenerative disorders (Olsen and Færgeman 2017; Pant et al. 2020; Yoon et al. 2022). In the case of AD and PD, sphingolipid metabolites have been both implicated as deleterious or protective. In the CSF and brain of AD patients, increased levels of ceramides were identified, potentially related to an increased activity of CERS enzymes. This ceramide elevation and the presence of ceramide‐enriched exosomes of amyloid β and phosphorylated Tau protein could potentiate plaque formation and spreading of the disease (Malm et al. 2016). Similar observations were described in PD patients (Olsen and Færgeman 2017; Dunn et al. 2019). Another example of the importance of lipid homeostasis in complex neurological disorders is related to DEGS1 variants associated with leukodystrophy. This pathology is defined by hypomyelination and neurodegeneration. DEGS1 is a member of the membrane fatty acid desaturase family, and mutations are recapitulated in several studies (Dolgin et al. 2019; Karsai et al. 2019; Pant et al. 2019). Moreover, several sphingolipid species and related enzymes have been implicated in peripheral neuropathies (Astudillo et al. 2015), amyotrophic lateral sclerosis (Bouscary et al. 2021), demyelinating disorders (Giussani et al. 2021), and Charcot Marie Tooth disease (Espinoza et al. 2023).

Another example of extensively studied pathologies is metabolic neuropathologies. A class of lysosomal storage disorders (LSDs), known as sphingolipidoses, is defined by an abnormal accumulation and storage of metabolites. Known pathologies such as Niemann‐Pick disease and Gaucher disease present different levels of abnormal sphingolipid metabolism, ranging from less complex metabolites, such as ceramides, to more complex sphingolipids, such as GM1. They can either arise from mutations present in the key enzymes of sphingolipid metabolism or from abnormalities in the lysosomal pathways (Platt 2014). A recent study by Jáñez Pedrayes et al. focused on another class of neurometabolic disorder, known as Congenital Disorder of Glycosylation (CDG), more specifically SLC35A2‐CDG (MIM#300896). It is an X‐linked dominant congenital disorder, arising from *de novo* mutations encoding for the UDP‐galactose transporter (*SLC35A2*). Symptoms have early developmental onset and are characterized by developmental delay, ID, severe epilepsy, hypotonia, and MRI abnormalities (cerebellar atrophy, delayed myelination, white matter lesions, and abnormal corpus callosum (Vals et al. 2019)). One class of severely impacted patients, exhibiting brain somatic mosaic variants, presents excess oligodendrocytes and heterotopic neurons in the subcortical white matter, increased oligodendroglial proliferation, and hypomyelination, known as MOGHE (mild malformation of cortical development with oligodendroglia hyperplasia in epilepsy). SLC35A5 is found in the Golgi apparatus and ER, transporting UDP‐galactose residues in order to modify glycans on glycoproteins and glycolipids. Patients present defects in transport and insufficient galactosylation of glycans. In this study, the authors showed that patient fibroblasts present an altered sphingolipid balance with an accumulation of glucosylceramides and a deficiency of digalactosylated glycosphingolipids and complex gangliosides. Moreover, they showed that galactose oral supplementation could improve clinical and biochemical symptoms in patients, as well as using serum‐derived hydroxylated GM3 as a biomarker of the disease (Jáñez Pedrayes et al. 2025). These findings provide insight into the understanding of complex pathologies linked to sphingolipid dysregulation, with promising diagnosis and therapeutic approaches.

Sphingolipids have been extensively studied in neurodegenerative and metabolic disorder patient conditions. However, little is known about their implications in neurodevelopmental functions and disorders. As mentioned previously, lipids are being increasingly described as key regulators of neuronal development, emphasizing the importance of understanding how abnormal lipid metabolism could lead to complex neurodevelopmental disorders in patients.

### 5.2 Ceramide Synthases and Epilepsy Syndromes

**FIGURE 5:**
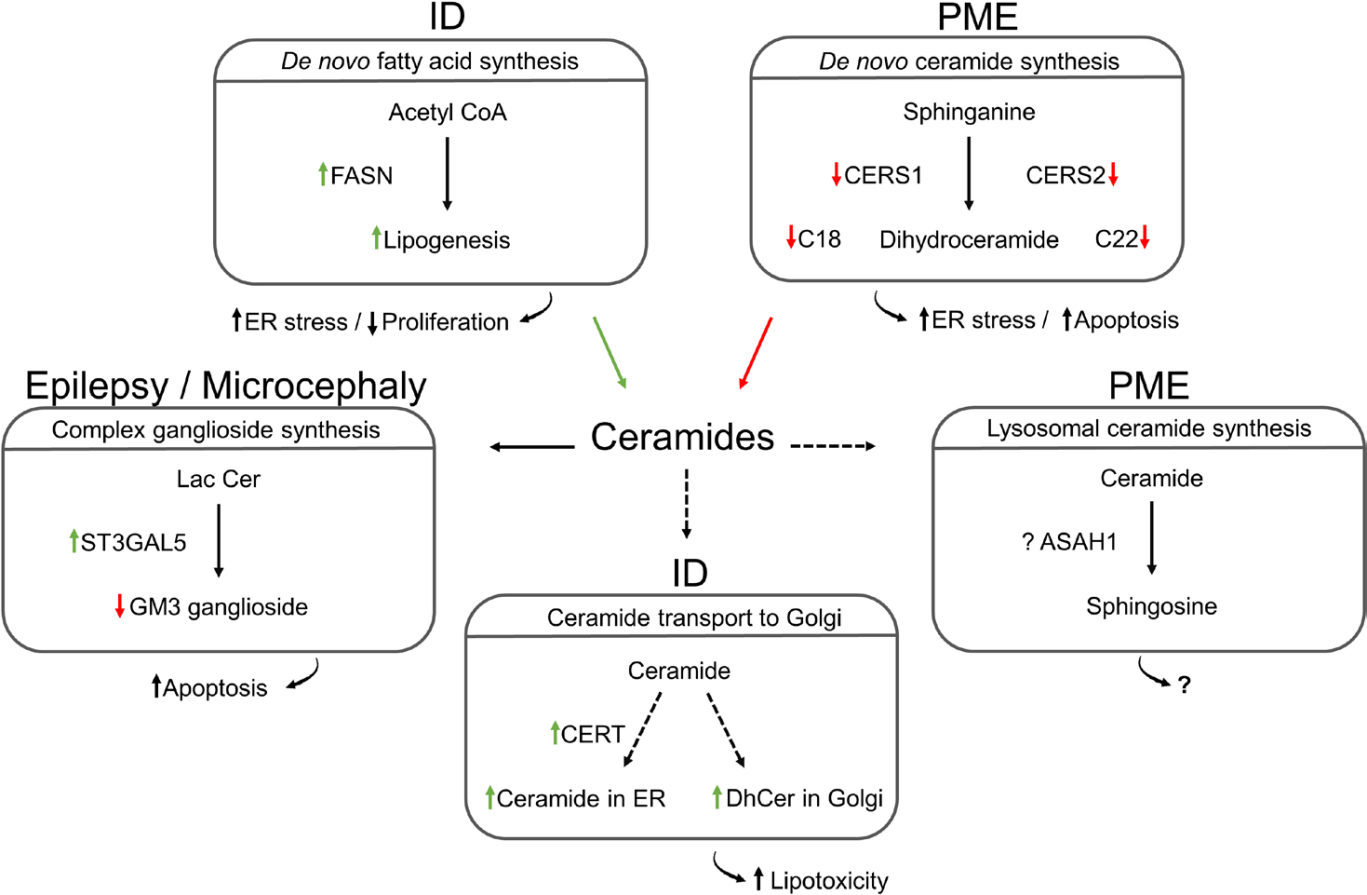
Neurological disorders related to changes in sphingolipids and ceramide levels. Several patient mutations in key enzymes of the sphingolipid pathway have been identified. These mutations will give rise to unbalanced levels of sphingolipid. Some have a direct impact on *de novo* ceramide synthesis and downstream metabolites, such as mutations in *CERSs* (Mosbech et al. 2014; Vanni et al. 2014) and *FASN* (Bowers et al. 2020) (upper panel). Mutations can also impact the synthesis of complex sphingolipids downstream of ceramide, thus, *ST3GAL5* mutations (centre left) will affect the production of complex gangliosides such as GM3. Mutations can affect other levels of ceramide production, such as in *ASAH1* (centre right) (Courage et al. 2021), present in lysosomes, mutations impacting ceramide production in other subcellular compartments. Mutations can also perturb ceramide transport and levels in the ER and Golgi apparatus. The CERT enzyme (lower panel) is involved in the transport of ceramides between the ER and Golgi apparatus. *CERT* mutations (Gehin et al. 2023) perturb this transport, which impacts upstream and downstream metabolite levels. All these changes in sphingolipid synthesis and localization will trigger lipotoxicity, which can lead to cellular dysfunction. Green arrow: Upregulation, Red arrow: Downregulation, Dotted black arrow: Indirect effect, Black arrow: Direct effect. DhCer, Dihydroceramide, ID, Intellectual disability; Lac Cer, Lactosylceramide; PME, Progressive myoclonic epilepsy.

Several studies have linked sphingolipid abnormalities to epilepsy. The described phenotypes are complex and often multifactorial, with neurodevelopmental and neurodegenerative features. We summarize predicted effects in Figure 5.

Epilepsy‐related patient mutations are found in different enzymes of the sphingolipid pathway. Some examples, such as *ASAH1* mutations, have been linked to progressive myoclonic epilepsy (PME) (Courage et al. 2021); the patients also display spinal muscular atrophy (Zhou et al. 2012). ASAH1 enzymes are found in lysosomes and convert ceramide to sphingosine. Thus, patients display epilepsy as well as lysosomal defects. Another enzyme, ST3GAL5, has been linked to infantile onset symptomatic epilepsy, also known as Amish epilepsy syndrome, with patients also exhibiting developmental stagnation and ID (Simpson et al. 2004). The enzyme is responsible for the production of complex gangliosides, essential for myelin sheath maintenance, by adding sialic acid to lactosylceramide. The CLN8 enzyme has been implicated in cases of progressive epilepsy with ID, also known as Northern epilepsy or Neuronal ceroid lipofuscinoses. The CLN8 enzyme is predicted to be involved in lipid synthesis, and lipidomic analyses of post‐mortem patient brains show a reduction in several lipids, such as ceramides, galactosyl, and lactosylceramide (Hermansson et al. 2005). These few examples emphasize the importance of lipid homeostasis in neurons, impacting different species that range from common lipid precursors to more complex sphingolipids in different cell compartments.

Focusing on ceramide homeostasis and epilepsy, studies have identified *CERS* mutations in patients with PME. This disorder is defined as neurodegenerative, as although the patient generally starts to display seizures during infanthood, there then follows a progressive cognitive deterioration even leading to dementia and ataxia. The first case reported in 2009 by Ferlazzo et al. was shown to impact 4 siblings, displaying a homozygote nonsynonymous mutation (c.549 C>G, NM_021267.3; p.H183Q, NP_067090) in *CERS1*. These patients display PME and progressive cognitive decline. The mutation was shown to impair CERS1 activity, since an altered ceramide profile was identified in patient fibroblasts by mass spectrometry (LC–MS/MS). The reduced enzymatic activity and C18‐ceramide level triggered an increased ER stress response and activation of pro‐apoptotic pathways (Ferlazzo et al. 2009; Vanni et al. 2014). Also, homozygous *CERS1* mutations (c.202 C>A (p.Leu68Met) and c.2010 G>A (p.Trp70*)) were found in two siblings, reported by Courage et al. The two patients also present PME, ataxia, and mild cognitive impairment (Courage et al. 2021). In these CERS1 cases, no impact on neurodevelopment is expected, since it is mainly expressed in mature neurons postnatally (Ginkel et al. 2012).

Defects linked to a deletion of the *CERS2* gene were reported by Mosbech et al. The patient has a *de novo* 27 kb heterozygous deletion on 1q21 encompassing the entire *CERS2* gene and displays PME, ataxia, and moderate ID. Of interest, at 27 years, minimal heterotopia (ectopic clusters of neurons) was found in the left temporal lobe of the patient. As for the CERS1 case, CERS2 activity was assessed in patient fibroblasts and found to be decreased, leading to a decrease in very long‐chain fatty acid ceramides and sphingolipids (Mosbech et al. 2014). The developmental aspect was not investigated in this patient, and this could be of great interest as CERS2 is expressed in the brain during embryogenesis and the patient displays two probable hallmarks of neurodevelopmental disorders, heterotopia and ID.

### 5.3 Microcephaly and ID

Neurodevelopmental disorders include malformations of cortical development (CMs), often associated with defective proliferation and migration during development. The major CM groups are linked to abnormal brain size (micro and megalencephaly), cortical folding (lissencephaly, polymicrogyria), and cellular localisation (heterotopias) (Romero et al. 2018). CMs are generally linked to neurological conditions such as epilepsy, autism spectrum disorder, ID, and developmental delay (Barkovich et al. 2012; Li et al. 2018). Little is known about lipid homeostasis in these pathologies. However, a few cases have already been linked to lipid deregulation, including microcephaly associated with ID. As mentioned previously, the ST3GAL5 enzyme was shown to be linked to infantile onset epilepsy in various patients. For 3 siblings with the p.E322K missense mutation, microcephaly was observed, associated with severe ID. The mutation was shown to lead to a tendency for an increased ST3GAL5 expression, causing an increased expression of downstream enzymes and an impact on the sphingolipid composition of patient cells. To determine the importance of this enzyme in the brain, a genetically modified zebrafish model was generated. Authors were able to replicate the results observed in patient cells and to show an increase of apoptosis in several brain regions, emphasizing the potential role of this enzyme in brain cell homeostasis (Boccuto et al. 2014). However, for this specific study, neurodevelopment was not investigated.

A well‐described case showing the importance of lipid availability during brain development and associated disorders links mutation in the FASN enzyme and ID (as mentioned in Section 2.3) (Bowers et al. 2020). Fatty acid availability is expected to impact ceramide composition. The homozygous variant *FASN R1819W* linked to ID is associated with increased activity in genetically modified *Fasn‐R1812W* mice and modified NSCs. Increased de novo lipogenesis triggers lipid accumulation and activation of the ER stress response, decreasing proliferation, as observed in the mouse model. The outcome of the mutation is thus likely a gain of function with an increased production of lipids, such as ceramides and sphingolipids (Bowers et al. 2020). Forebrain‐specific mutant mice display severe microcephaly due to abnormal progenitor behavior (Gonzalez‐Bohorquez et al. 2022). Also, a consortium showed evidence of a potential link between a *FASN* variant and epileptic encephalopathies (Appenzeller et al. 2014). These studies highlight the importance of lipid availability in key cell types during neurodevelopment, such as progenitor cells. If these lipids are dysregulated, neurodevelopmental defects appear in mouse models and human patients.

### 5.4 Lipid Transport Protein CERT, an ID Disorder

Another enzyme involved in sphingolipid availability is linked to ID. In this case, the enzyme is not involved in the synthesis of sphingolipids but in the transport of ceramides. Mutations have been found in different individuals who display complex multifactorial pathologies, involving both neurodevelopmental abnormalities and neurodegeneration, ranging from ID, seizures, and leukodystrophy, which complicates the understanding and potential causality link between the mutation and the observed abnormalities. CERT enzymes are present at the interface between the ER and the trans Golgi (Figure 1), allowing the specific transport of ceramides from these cellular compartments in order to produce complex sphingolipids, such as sphingomyelin. Once the correct amount of sphingomyelin is reached, the enzyme is phosphorylated and changes conformation, which inactivates its catalytic activity. The enzyme is composed of four different domains. The Pleckstrin homology domain is responsible for the interaction with the trans Golgi, the SRR (serine‐rich region) domain is targeted by protein kinase D for its phosphorylation, the FFAT (2 phenylalanines in an acidic tract) motif, which interacts with VAP‐A and VAP‐B in the ER, and the START domain, which extracts ceramides from the ER membrane. 87% of the mutations are found in these specific domains with different phenotype severities (Gehin et al. 2023). The most severe phenotypes are correlated with mutations in the SRR domain, triggering a hyperphosphorylation of the enzyme leading to an increased activation of mutant CERT with a gain of function effect (Murakami et al. 2021; Tamura et al. 2021). The hyperactivation triggers an increase in *de novo* sphingolipid synthesis with a decreased ceramide availability at the ER, which reduces serine palmitoyltransferase (SPTLC) inhibition and increases dhCer in the trans Golgi, potentially producing excess sphingolipids, which ultimately can be neurotoxic. These studies were focused on understanding the outcomes of the mutation on enzymatic activity and potential cellular responses. The effect of this imbalance in sphingolipid composition and availability in different cellular compartments and the link with the observed patient phenotypes is still to be explored. Gehin et al. generated a Drosophila model with a CERT gain of function and showed decreased brain and head size, changed lipidomic profiles in the brain, and motor abnormalities. These defects were reversed by the pharmacological inhibition of CERT (Gehin et al. 2023). Tamura et al. hypothesized that a proper balance between GalCer and sphingomyelin could be perturbed, implying an instability of the myelin sheath, potentially linked to leukodystrophy (Tamura et al. 2021). These patient cases with differential phenotype severity correlated with the potential outcome of the mutation show that sphingolipid balance is essential for brain homeostasis.

### 5.5 Neuropsychiatric Disorders

Other neurodevelopmental disorders have been linked to sphingolipid imbalances, similar to neurodegenerative disorders such as AD (Matanes et al. 2019). Emerging evidence suggests the significance of lipid regulation in disorders such as autism spectrum disorder (ASD) (Yui et al. 2022; Puljko et al. 2025). In children with ASD, increased serum levels of S1P were observed. Sphingosine kinase (SPHK) enzymes catalyze the production of S1P from sphingosine in different cell compartments. S1P is an active metabolite abundant in brain tissue, with a role in neurodevelopment, acting on proliferation, survival, and apoptosis. Two Sphk enzymes are found in different cell compartments with differential roles in cell survival. Sphk1 is found at the cell membrane and is pro‐proliferative and pro‐survival, whereas Sphk2 is found in mitochondria and promotes apoptosis. Using the valproic acid rat model of autism‐like phenotypes, Sphk2 and S1P were found to be upregulated. The rats displayed learning and memory impairments, with apoptosis of hippocampal neurons. Pharmacological inhibition of Sphk was able to reduce S1P levels and decrease the observed phenotype (Wu et al. 2018). The origin of increased S1P serum levels in ASD patients is yet to be discovered. Recently, Yan et al. conducted a metabolomic study with CSF from ASD patients to study autistic regression, which relates to the loss of previously acquired neurodevelopmental skills. ASD patients with autistic regression showed dysregulation of sphingolipid pathways, such as increased S1P levels and elevated ceramides, hexosylceramides, sphingosines, and sulfatides, whereas sphingomyelin tended to decrease (Yan et al. 2025). The mechanisms underlying such changes are yet to be studied and understood. The use of metabolomics studies helps to identify biomarkers of poorly diagnosed and understudied clinical symptoms in ASD, such as clinical regression, gaining potential insights for future therapeutic approaches.

Lastly, a few studies have reported changes in sphingolipid composition of postmortem brain tissues from schizophrenia patients. Observed changes in ceramide and sphingomyelin levels were identified, with changed expression of SPTLC and ASAH1 enzymes, associated with perturbed myelination (Mühle et al. 2013). Several enzymes involved in the sphingolipid pathway were also shown to be decreased in the prefrontal cortex from patients (Narayan et al. 2009). Also, two single nucleotide polymorphisms within *ASAH1* were significantly associated with schizophrenia (Zhang et al. 2012). Psychiatric disorders are complex neurodevelopmental pathologies with multiple origins (potentially gene/environment), and little information is available concerning sphingolipid homeostasis and such disorders, with only up until now a few reported cases.

### 5.6 Diagnostics and Treatment

Sphingolipids are starting to be of interest for the diagnosis and treatment of various pathologies. Until now, this has mainly been studied in disorders such as cancers and obesity. Such strategies could be implemented in neuropathological disorders in the future. For example, drugs are already being used in patients to inhibit sphingolipid enzymes (Jamjoum et al. 2023). As well, sphingolipids could be of interest as biomarkers of diseases as they are present in the blood or CSF of patients where lipid profiles can be generated (Matanes et al. 2019). As mentioned previously, changes in sphingolipid composition have been observed in patients with different pathologies. Therefore, the ultimate goal would be to be able to use sphingolipids for pro‐diagnosis and to be able to restore sphingolipid levels with different strategies in these patients. At present, some drugs are used in patients such as inhibitors, but often they are not specific to a particular member of an enzyme family and have various targets. Furthermore, no drugs have been developed to enhance enzymatic activity, and strategies such as the delivery of specific sphingolipid species should be explored.

## 6 Conclusion

We summarize here the importance of lipids not only as bioenergetic fuel substrates, but also in the maintenance of membrane integrity and regulation of different signaling pathways essential for cortical development. The presence of ceramide‐based lipids in different intracellular compartments further emphasizes their importance in the maintenance of the structural integrity of different organelles that are highly critical for proper cerebral cortex development. Although many studies described here have been performed in different cell types that are also relevant for embryonic NSCs, the significance of these lipid‐based processes in the context of neurodevelopment remains understudied. The alteration of ceramide‐based lipids in different neurological disorders illustrates the additional importance of understanding their functional relevance in normal developmental processes. Are lipid changes a cause or consequence of the disorder? This remains an open question. Lipid dysregulation could cause impaired downstream processes that may be currently unknown, or in some cases, as described, the build‐up of substrates could cause ER stress and apoptosis upon lipotoxicity. It is thus necessary to carefully decipher the dysregulated processes in the context of disease for devising better treatment options in the future.

## Author Contributions

**Kaviya Chinnappa:** conceptualization, writing – original draft, writing – review and editing. **Fiona Ballorin:** conceptualization, writing – original draft, writing – review and editing. **Fiona Francis:** conceptualization, funding acquisition, supervision, writing – original draft, writing – review and editing.

## Consent

Informed consent was achieved for all subjects, and the experiments were approved by the local ethics committee.

## Conflicts of Interest

The authors declare no conflicts of interest.

## Data Availability

Data sharing not applicable.
